# Metastasis of differentiated thyroid cancer in the subchondral bone of the femoral head: a case report

**DOI:** 10.1186/s12891-015-0748-2

**Published:** 2015-10-09

**Authors:** Naoki Mizoshiri, Toshiharu Shirai, Ryu Terauchi, Shinji Tsuchida, Yuki Mori, Masazumi Saito, Keiichiro Ueshima, Toshikazu Kubo

**Affiliations:** Department of Orthopaedics, Graduate School of Medical Science, Kyoto Prefectural University of Medicine, Kamigyo-ku, Kyoto 602-8566 Japan

**Keywords:** Thyroid cancer, Bone metastases, Osteonecrosis of the femoral head, Subchondral bone metastases

## Abstract

**Background:**

Differentiated thyroid cancer (DTC) is relatively rare and can metastasize to both the lungs and bones. The great majority of bone metastases occur in red marrow regions where blood flow is high. Only one patient has been described with direct DTC metastasis to the subchondral bone of the femoral head.

**Case presentation:**

The patient was a 68-year-old Japanese female who had presented with left hip joint pain at age 63 years. At age 51 years, she had been diagnosed with DTC and underwent partial excision. X-rays showed partial femoral head collapse, suggesting osteoarthritis or idiopathic necrosis of the left femoral head. Three years later, a ^131^ I whole-body scan showed accumulation in the left femoral head, resulting in a diagnosis of DTC metastasis to the left femoral head. Bipolar hip arthroplasty was performed. Examination of the excised femoral head resulted in a final diagnosis of metastasis of follicular thyroid cancer, which was limited histopathologically to the subchondral bone of the femoral head.

**Conclusion:**

Tumor metastasis to the subchondral bone of the femoral head is exceedingly rare. Overall survival of patients with bone metastasis is improved by complete resection. Differential diagnosis of patients with a previous history of DTC who present with femoral head collapse should include bone metastasis of DTC.

## Background

Thyroid cancer is a relatively rare disease, accounting for approximately 1 % of all malignant neoplasms, about 0.5 % in men and 1.5 % in women [1]. Various types of thyroid cancer have been identified, including differentiated thyroid cancer (DTC), Hürthle-cell cancer, undifferentiated carcinoma and medullary carcinoma. About 90 % of thyroid cancers are DTC, including both papillary (70–75 %), and follicular (15–20 %) cancers. Papillary DTC is characterized by indolence and localized spread, but may metastasize to the lungs and bones. Follicular cancer is known to preferentially metastasize to the lungs and bones [2]. Bone metastases have been reported in 2.3–12.7 % of patients with DTC [3]. Most bone metastases occur in areas of high blood flow, including the red marrow regions of the axial skeleton, including the vertebrae (42–52 %), femur (9–20 %), skull (2–16 %) and pelvis (5–13 %) [4, 5].

Blood for the femoral head is supplied by branches of the profunda femoris artery, e.g. the lateral and medial circumflex femoral arteries. Furthermore, there is little collateral circulation, resulting in ischemic necrosis. Bone metastasis were reported difficult to distinguish from osteonecrosis or arthritis on magnetic resonance imaging (MRI) and fluorine-18 fluorodeoxyglucose whole-body positron emission tomography (FDG-PET) [6]. The most common site of femur metastasis in various cancers, including thyroid cancer, is the femoral neck (50 %), Followed by the subtrochanteric (30 %) and intertrochanteric (20 %) regions [7, 8]. To date, no patient with breast cancer has shown a single metastasis to the femoral head alone; rather, patients with femoral head metastasis had more than five metastasic lesions each [9]. Thyroid cancer metastasis to the subchondral bone of the femoral head is exceedingly rare, with only one previous patient described [10]. This report describes a patient with a thyroid cancer metastasis to the subchondral bone of the femoral head.

## Case presentation

The patient was a 68-year-old Japanese female who had presented with left hip joint pain at age 63 years. At age 51 years, she had been diagnosed with thyroid cancer and underwent partial excision, with the pathological diagnosis being a follicular DTC. At age 62 years, she was diagnosed with cancer in the right kidney, which was totally excised.

**Fig. 1:**
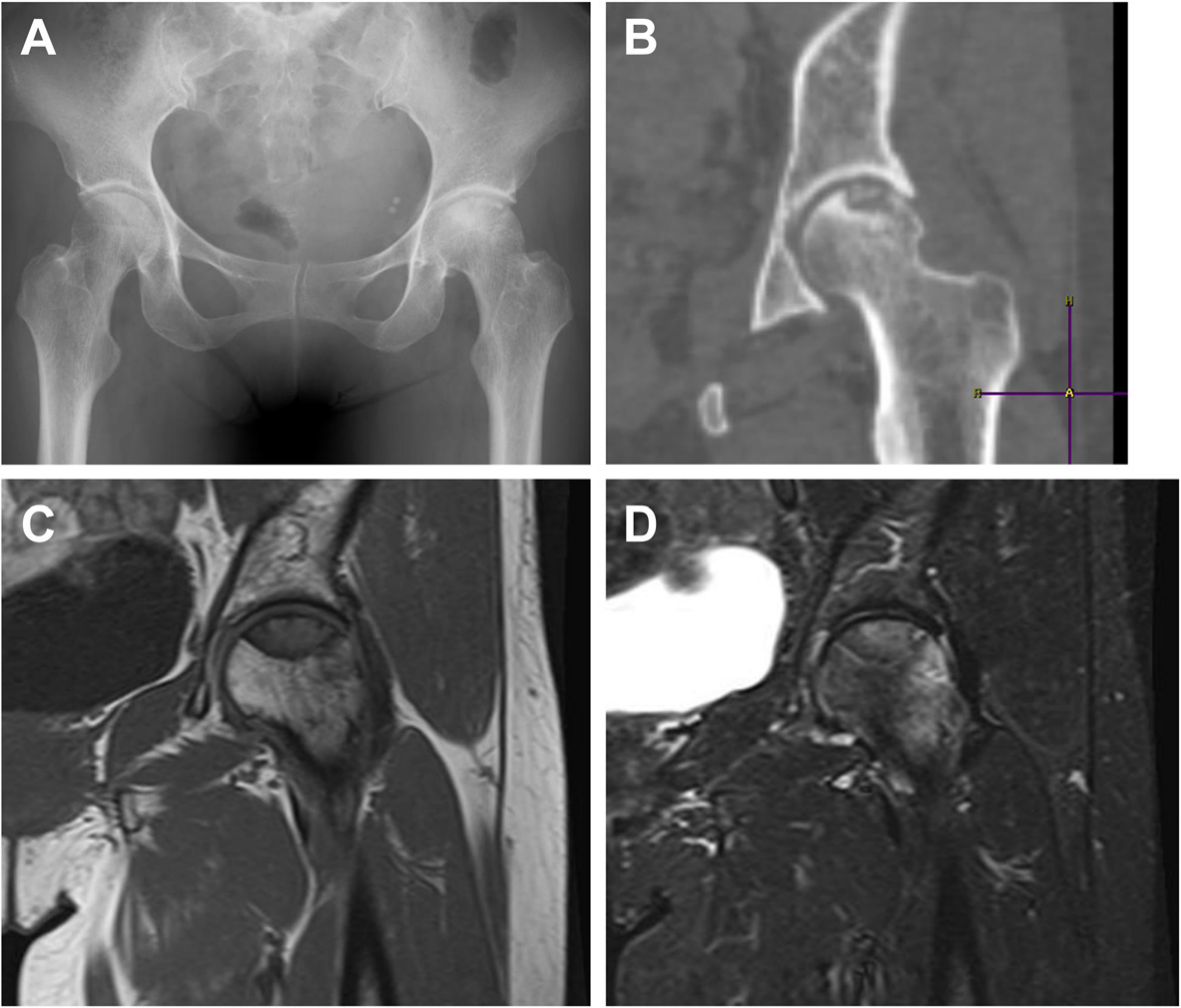
**a** X-ray of the hip joint showing left partial femoral head collapse. **b** CT image showed a lesion in the loaded part of the left femoral head. **c** Coronal T1 weighted image (T1WI) on MRI, showing low intensity of the left femoral head lesion. **d** Coronal short tau inversion recovery (STIR) on MRI, showing partially high intensity of the femoral head lesion

**Fig. 2:**
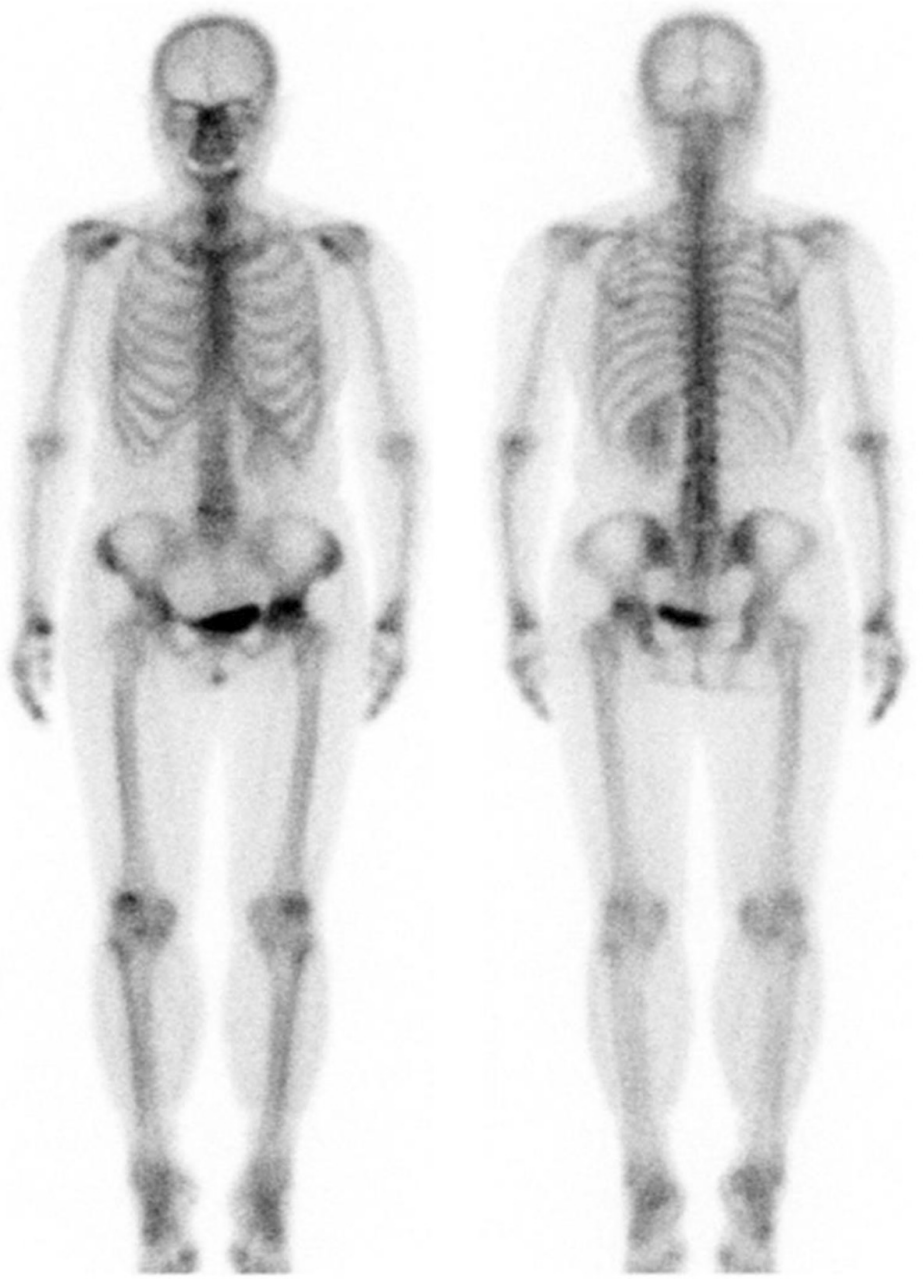
Bone scan showing that the only metastatic lesion was in the femoral head

**Fig. 3:**
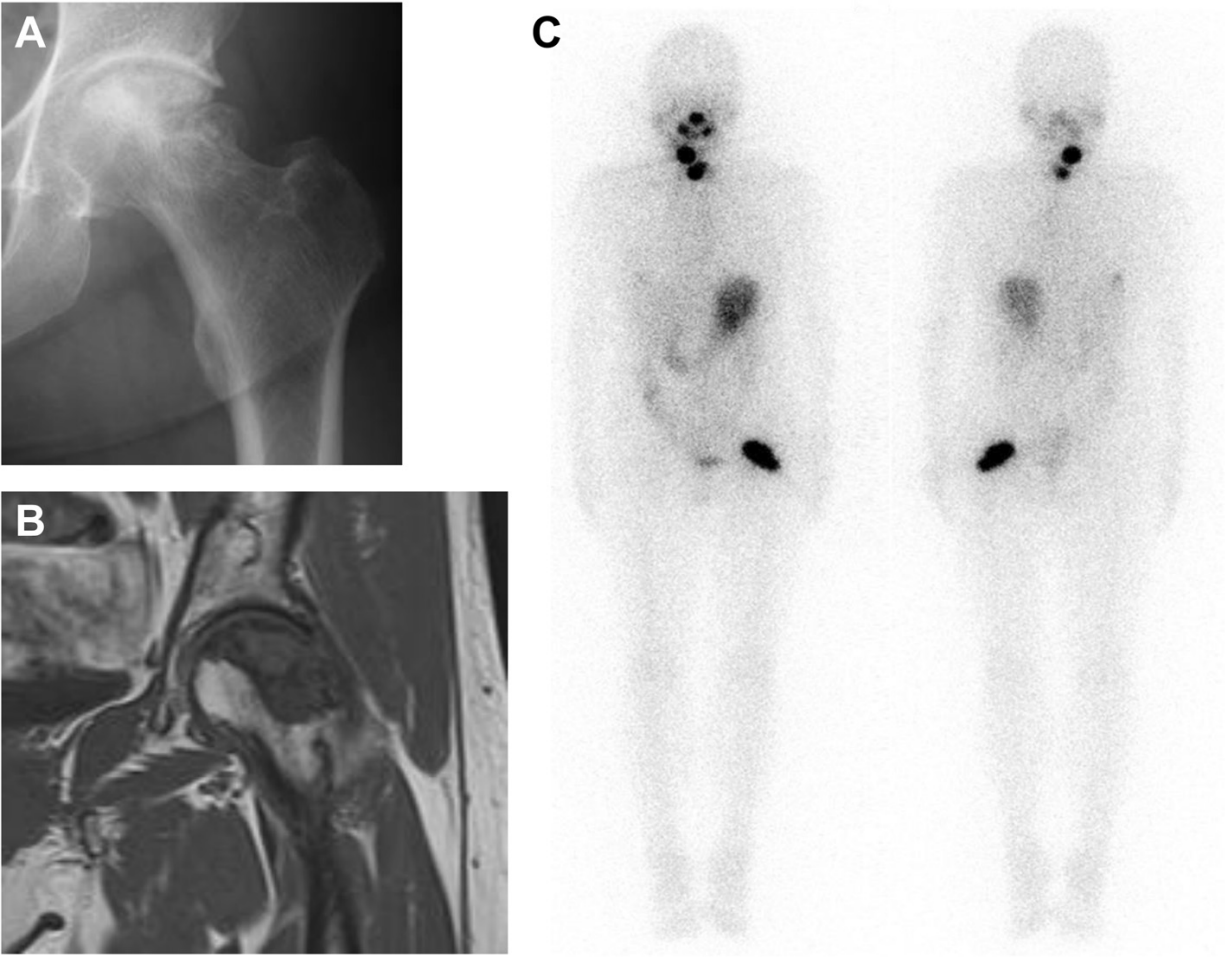
**a** X-ray of the hip joint 3 years after initial consultation, showing that the collapsed area had expanded. **b** Coronal T1 weighted MR image showing expanded low intensity of the left femoral head lesion. **c** ^131^ I whole-body scan (WBS) showing accumulation of radioactivity in the left femoral head

**Fig. 4:**
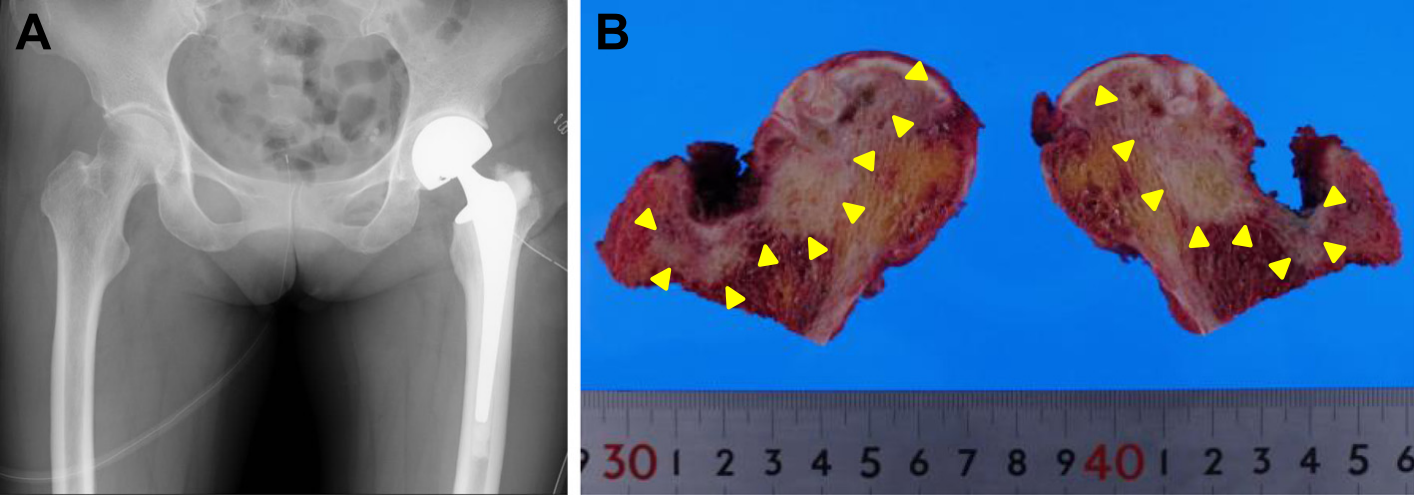
**a** X-ray, showing the left hip joint after BHA. **b** Photograph of the vertically divided left femoral head after surgery. DTC was observed in the subchondral region of femoral head (*arrowhead*). There was no cartilage discontinuity

At her first visit for hip pain, physical examination of the hip and blood tests showed no abnormal findings. However, X-rays (Fig. 1a) and computed tomography (CT) (Fig. 1b) showed partial femoral head collapse. On MRI, the focal lesion of the femoral head had a band-like shape, with low intensity on T1WI and high intensity on STIR, (Fig. 1c, d). Bone scans showed no accumulation of radioactivity, except for the femoral head (Fig. 2). The patient was differentially diagnosed with osteoarthritis of the hip or idiopathic necrosis of the femoral head. Because she had slight pain in the hip joint, she was followed up by X-rays of her hip joint. Three years after the initial consultation, a bone metastasis was observed in her right rib and was excised. The excised tumor was diagnosed histopathologically as a metastasis of follicular thyroid cancer. Subsequently, X-rays (Fig. 3a) and MRI (Fig. 3b) showed that the lesion and the area of collapse had expanded, with a ^131^ I whole-body scan showing accumulation of radioactivity in the left femoral head (Fig. 3c). These findings suggested that the lesion in the femoral head was a metastasis of thyroid cancer. The patient underwent bipolar hip arthroplasty (BHA) (Fig. 4a), with the excised femoral head diagnosed as a metastasis of follicular thyroid cancer. Histopathologic examination showed that the cancer cells were limited to the subchondral bone of the femoral head (Fig. 4b). The day after surgery, the patient was able to walk with full-weight-bearing. At present, 18 months after BHA, there has been no evidence of additional metastatic lesions and her Musculoskeletal Tumor Society (MSTS) score [11] is 100 %.

## Conclusions

DTC is one of the most curable cancers [12]. DTCs are characterized by a slowly progressive course, and have a 10-year survival rate of 80–95 % [2]. However, the occurrence of distant metastases reduces the overall 10-year survival rate to 40 % [13]. Previous studies have reported that 25 % of metastases were to the bone, 49 % to the lung and 15 % to both. Bone metastases have been reported in 2–13 % of patients with DTC, being significantly more frequent in patients with follicular cancer (7–28 %) than in those with papillary cancer (1.4–7 %) [13–15]. The patient described here had follicular cancer. Patients with thyroid cancer and bone metastases have a poor prognosis, with 10-year survival rates ranging from 0–34 % [16]. Complete resection of bone metastases of DTC has been associated with a significant improvement in survival [3, 17].

Most bone metastases of DTC occur in regions of high blood flow, including the vertebrae (42-52 %), femur (9–20 %), skull (2–16 %) and pelvis (5–13 %) [4, 5]. Anatomically, bone metastasis to the femoral head is very rare, with direct involvement of the subchondral region reported in only one previous patient [10]. Diagnosis is therefore very difficult, with these lesions appearing similar on imaging modalities to osteonecrosis and osteoarthrosis.

Bone metastases of thyroid cancer may be accompanied by pain, but often are clinically silent, making them difficult to detect. Imaging modalities, including whole body bone MRI, CT, bone scan, ^131^ I whole-body scan (WBS) and FDG-PET, should be performed in patients with suspected bone metastases of DTC. Whole body MRI provides detailed images of both bone and bone marrow. CT can evaluate the extent of metastatic lesions and is especially useful for sites that are difficult to evaluate. If whole body MRI or CT detects a bone metastasis, a directed MRI or CT scan should be employed to specifically define the lesion of interest and aid in the planning of surgery or radiotherapy or the use of novel modalities to treat destructive osseous metastases. Bone scans with ^99m^Tc diphosphonate or methylene diphosphonate (MDP) are most frequently used for localization and staging. ^131^ I-WBS is more specific and sensitive than a bone scan but only for well differentiated thyroid cancer [4]. These scans have low sensitivity in detecting distant metastases from thyroid cancer and in localizing bone metastases. In contrast post-treatment ^131^ I-WBS may be highly sensitive (61–65 %) in detecting osseous lesions, as in the patient described here. Because ^131^ I-WBS can detect some, but not all, bone metastases, further testing is needed [16]. For example, FDG-PET shows preferential tracer uptake by malignant cells with a high turnover rate due to increased glucose metabolism. FDG-PET is useful in patients with metastatic poorly differentiated thyroid cancer, in those with high thyroglobulin levels and in patients negative on ^131^ I-WBS [18]. X-rays and CT scans in the patient described here showed femoral head collapse, whereas MRI showed that the focal lesion of the femoral head had the shape of a band, with low intensity on T1WI and high intensity on STIR. Furthermore, bone scans showed no other metastatic lesions. The differential diagnosis included osteoarthritis of the hip and idiopathic necrosis of the femoral head. Three years later, a metastasis in the right rib was detected, with ^131^ I-WBS showing the metastasis to the left femoral head. Because the overall survival of patients with bone metastasis is improved by complete resection, early diagnosis and treatment is important. Bone metastasis of DTC should be considered in the differential diagnosis of patients with a previous history of DTC and femoral head collapse.

## Consent

Written informed consent was obtained from the patient for publication of this case report and accompanying images. A copy of the written consent is available for review by the Editor-in-Chief of this journal.

## Abbreviations

DTC: Differentiated thyroid cancer
CT: Computed tomography
MRI: Magnetic resonance imaging
FDG PET: 2-deoxy-2-[fluorine-18]fluoro-D-glucose integrated with computed tomography
^131^ I-WBS: ^131^ I whole-body scan
BHA: Bipolar hip arthroplasty
